# Anodal transcranial direct current stimulation associated with aerobic exercise on the functional and physical capacity of patients with heart failure with reduced ejection fraction: ELETRIC study protocol

**DOI:** 10.1186/s13063-023-07694-2

**Published:** 2023-11-17

**Authors:** Vanessa Christina Costa da Silva, Fernando Zanela da Silva Arêas, Antônio Luiz Ribeiro Boechat Lopes, Fernando Fonseca de Almeida e Val, Allyson Guimarães da Costa, Joana Colares Correa dos Santos, João Marcos Bemfica Barbosa Ferreira, Guilherme Peixoto Tinoco Arêas

**Affiliations:** 1 Graduate Program In Basic And Applied Immunology, Instituto de Ciências Biológicas, Universidade Federal do Amazonas, Avenida General Rodrigo Octavio Jordão Ramos, 1200 - Coroado I, Manaus, Amazonas 69067-005 Brazil; 2 Center of Health Sciences, Discipline of Physiotherapy, Universidade Federal do Espírito Santo, Vitória, Espírito Santo, Brazil; 3 Graduate Program in Tropical Medicine, Universidade do estado do Amazonas, Manaus, Amazonas Brazil; 4 Graduate Program In Human Movement Sciences, Universidade Federal do Amazonas, Manaus, Amazonas Brazil

**Keywords:** Clinical Trial, Heart failure, Exercise training, Transcranial direct current stimulation

## Abstract

**Background:**

The hallmark symptom of heart failure (HF) is severe exercise intolerance. Fortunately, accumulated evidence suggests that exercise programs improve physical performance, enhance autonomy in daily activities and quality of life, and reduce cardiovascular and other hospitalizations. Recently, experimental studies have explored the application of non-invasive brain stimulation techniques, especially transcranial direct current stimulation (tDCS), aiming to improve physical performance due to its ability to modulate brain functioning. The primary objective of the present study is to evaluate the effects of anodal tDCS associated with aerobic exercise on the functional capacity of patients with HF with reduced ejection fraction (HFrEF). Secondary objectives are to compare the effects of tDCS associated with aerobic exercise *vs*. sham-tDCS associated with aerobic exercise on cardiopulmonary exercise capacity; inflammatory cytokines; and quality of life.

**Methods:**

This is a two-arm, prospectively registered, randomized trial with concealed allocation, double-blind, and intention-to-treat analysis. Forty-four patients with HFrEF will be recruited. The experimental group will undertake 25–30 min aerobic exercise training associated with tDCS, for 4 weeks. The control group will undergo the same aerobic exercise training, but with sham-tDCS. The primary outcome will be functional performance by the 6-min walk test. Secondary outcomes will include cardiopulmonary exercise capacity, inflammatory cytokines, and quality of life. Outcomes will be collected by a researcher blinded to group allocation at baseline (T0) and after 4 weeks of intervention (T1).

**Discussion:**

Although previous studies have investigated the combined effect of tDCS on T3 area and physical performance and have suggested that tDCS could have reduced ratings of perceived exertion by affecting the activity of the insular cortex, and therefore increase exercise tolerance, this study is the first to evaluate the effects of the addition of anodal tDCS to aerobic exercise training for improving physical and functional performance, decreasing the perceived exertion, altering the quantification of inflammatory cytokines, and improving the subclinical values of the cardiopulmonary test in patients with HFrEF, which could result in an important advance in cardiac rehabilitation for patients with chronic HF.

**Trial registration:**

Brazilian Registry of Clinical Trials (ReBEC) RBR-10w787j6. Registered on 25 April 2023. https://ensaiosclinicos.gov.br/pesquisador

## Background

Currently, cardiovascular diseases constitute the main group of diseases in causes of death worldwide and are responsible for premature deaths, loss of quality of life, and biological and social impacts [13, 15, 28]. Heart failure (HF) is a common terminal symptom of many cardiovascular diseases [11], and as part of this group, it is related to high direct hospitalization costs and indirect costs, with a high burden of morbidity for patients [27, 33].

The hallmark symptom of HF is severe exercise intolerance, which is multifactorial and attributed to central and peripheral pathophysiological mechanisms, such as impaired cardiorespiratory reserve, excessive systemic vascular resistance, impaired vasodilation capacity, abnormal redistribution of blood flow and muscle perfusion, and decreased mitochondrial volume density and skeletal muscle function [32]. Heart injury causes an increase in the neurohumoral activity of some systems, such as the sympathetic nervous system, which triggers inflammation and oxidative stress, culminating in several alterations in the musculoskeletal system, including a reduction in strength [17]. Elevated levels of interleukin-6 (IL-6) and tumor necrosis factor (TNF-alpha) were described as important factors for the progression of chronic HF [10, 17]. Exercise intolerance, chronic fatigue, and inability to perform activities are the main symptoms of HF and are associated with poor quality of life and adverse outcomes [3, 8, 14, 33].

Fortunately, accumulated evidence suggests that exercise programs improve physical performance, lessen left ventricular remodeling, improve autonomy in daily activities and quality of life, and reduce cardiovascular and other hospitalizations [4, 11, 13, 16, 23, 25, 32]. Based on principles of exercise physiology, performance depends on the integrative response of the pulmonary, circulatory, and musculoskeletal systems. Goes-Santos et al. [11] suggest that there is a link between an increase in physical capacity and amelioration in neurovascular control and skeletal myopathy in exercise-trained patients with HF with reduced ejection fraction (HFrEF). Peripheral adaptations, particularly in skeletal muscle, are the primary mechanism for increasing maximal oxygen uptake (*V̇*O_2_max) after exercise training in patients with HF, and the reason may be that, compared with cardiac muscle, skeletal muscle is more plastic and has the potential for rapid, large improvements in function after even a brief period of exercise [28].

Recently, experimental studies have explored the application of noninvasive brain stimulation techniques, especially transcranial direct current stimulation (tDCS), aiming to improve physical performance due to its ability to modulate brain functioning [2, 18, 21]. Even more interestingly, tDCS may have a moderate positive impact on performance levels [30], and both tDCS and aerobic exercise are capable of modulating cognitive functions, hemodynamic activity, brain activity, and perceived exertion in submaximal exercise [19, 31]. A possible target for tDCS is applying over the left temporal cortex to modulate the insular cortex (IC), which is implicated in the control of cardiac autonomic function, resulting in increased parasympathetic modulation at rest and during exercise, and delaying the rate variability threshold would increase the time exercising with a lower cardiovascular load, which could postpone fatigue [12, 19]. Okano et al. [24] and Montenegro et al. [20] studied the effects of anodal tDCS in IC and observed a decrease in dyspnea and fatigue and faster recovery after exercise in athletes in their trials.

**Fig. 1:**
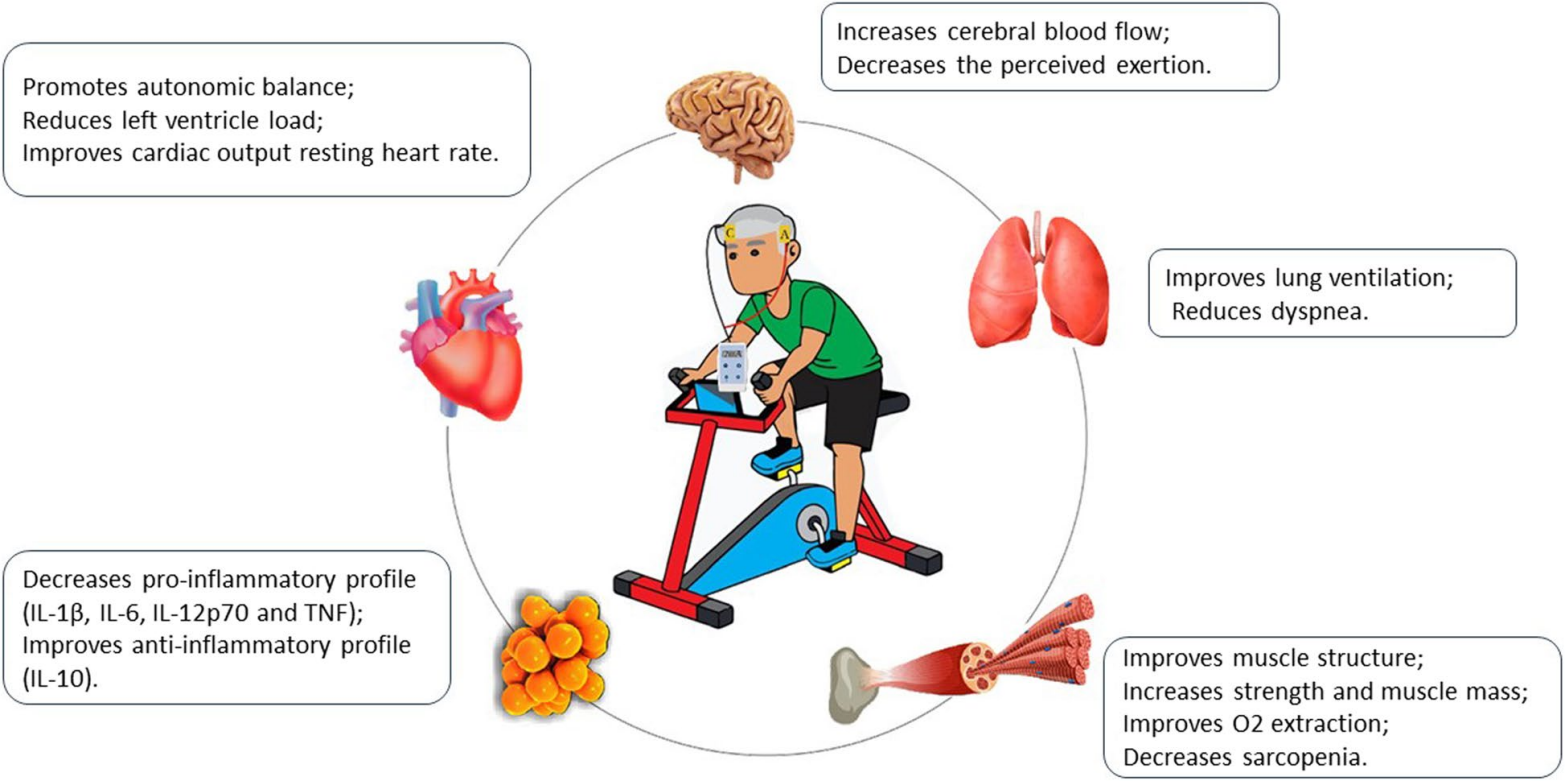
Illustration of the benefits of the intervention

Based on these studies, we hypothesized that patients with HFrEF who received anodal tDCS in IC associated with aerobic exercise, when compared to those submitted to sham-tDCS associated with aerobic exercise, will have better physical and functional performance, decreasing perceived exertion, altering the quantification of inflammatory cytokines, and improving the subclinical values of the cardiopulmonary test (*V̇*E/*V̇*CO2 slope and OUES), thus innovating in terms of therapeutic possibilities for so many patients with the same pathological condition in Brazil and worldwide (Fig. 1). The primary objective of the present study is to evaluate the effects of anodal tDCS associated with aerobic exercise on the functional capacity of patients with HFrEF. Secondary objectives are to compare the effects of tDCS associated with aerobic exercise *vs*. sham-tDCS associated with aerobic exercise on (1) cardiopulmonary exercise capacity, (2) inflammatory cytokines, and (3) quality of life.

## Methods

### Study design

The ELETRIC study is designed to investigate the effects of noninvasive brain stimulation in patients with HF (*Eletroestimulação em Insuficiência Cardíaca*, in Portuguese).

**Fig. 2:**
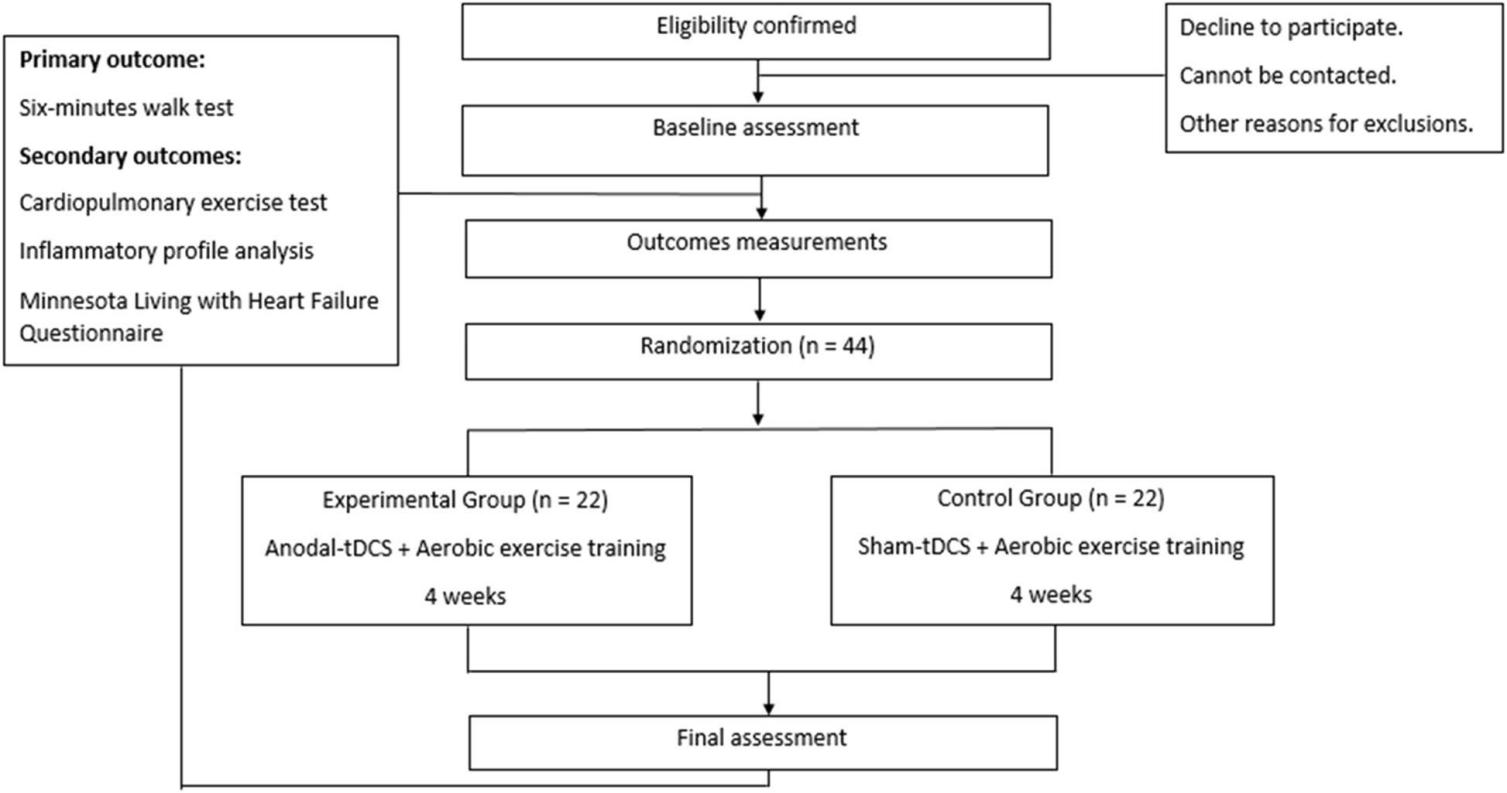
Proposed trial design

A clinical, randomized parallel-design 1:1 ratio allocation, controlled, blinded assessors, participants and therapists, and intention-to-treat analysis will be carried out by the Federal University of Amazonas (Universidade Federal do Amazonas) at the University Hospital Getúlio Vargas, in the city of Manaus, North Brazil (Fig. 2). The study was approved by the ethics committee (CAAE 64652122.0.0000.5020) and registered by ReBEC (Brazilian Registry of Clinical Trials), registry RBR-10w787j6. The study protocol adheres to the SPIRIT 2013 recommendations.

### Eligibility criteria

The volunteers will be included in the study if they meet the following:

- Adult men or women
- Age equal to or older than 18 years old
- Clinically stable HF (ischemic or nonischemic) for at least 3 months before randomization
- Left ventricular ejection fraction of less than 40% evaluated by echocardiogram
- Functional class II or III by New York Heart Association
- Optimized pharmacological treatment

The volunteers will be excluded if they present the following:

- Stable and unstable angina
- Severe cardiac arrhythmias
- Advanced atrioventricular block (second and third degree)
- Symptomatic coronary artery disease
- Pacemaker or implantable ventricular defibrillators
- Decompensated HF less than 3 months
- Metabolic syndromes linked to the thyroid or liver
- Body mass index (BMI) less than 18.5 and greater than 34
- Acute myocardial infarction and/or cardiac surgery for less than 6 months
- Uncontrolled diabetes, high blood pressure, and dyslipidemia
- Diagnosed lung disease, such as exercise-induced asthma and pulmonary fibrosis
- Orthopedic or musculoskeletal disorders that limit physical exercise (such as osteoarthritis in the lower limb and/or spine)
- Neurological and/or cranial alterations that prevent the application of tDCS
- Diagnosed seizures
- Regular practice of physical exercise in the last 12 months
- Pregnant
- Smokers
- Alcoholics
- Impaired cognitive status that compromises the understanding of the steps and completion of the study protocol, screened by the Mini-Mental State Examination (MMSE). The cutoff scores are 26 for people with high levels of education, 18 for people with elementary and middle levels, and 13 for illiterate people.

### Initial assessment

One of the researchers (VCCS), in a reserved room at the University Hospital Getúlio Vargas, will explain the study and deliver the informed consent form with time for the patient to think and respond about his/her participation.

Additionally, the consent form will inquire whether participants consent to the utilization of their data in the event of their withdrawal from the trial. Participants will also be requested to grant permission for the research team to share pertinent data with individuals affiliated with the participating universities or regulatory authorities, as applicable. It is important to note that this trial does not involve the collection or storage of biological specimens.

Volunteers will undergo an initial assessment, which will collect information regarding the current illness, past illness, comorbidities, drugs in use along with dosage, systolic (SBP, mmHg) and diastolic (DBP, mmHg) blood pressure, heart rate (HR, bpm), respiratory rate (RH, rpm), body composition (weight, height, and BMI), peripheral oxygen saturation, and perception of exertion by Borg CR-10.

Cardiac function will be assessed by two-dimensional transthoracic echocardiography supplemented with M-mode, pulsed, tissue, and color Doppler, according to the recommendations of the American Society of Echocardiography (American Society of Echocardiography, 2005). Measurements of the cardiac cavities and evaluation of ventricular systolic and diastolic functions will be performed. The cavities measured will be left ventricular diastolic diameter (LVDD), right ventricular diastolic diameter (RVDD), and left atrial volume indexed by body surface (LAEi). Ventricular systolic function will be assessed by capturing left ventricular ejection by Simpson’s method (LVEF), by the systolic velocity of the lateral mitral annulus (lateral S′), and by the systolic velocity of the tricuspid annulus (tricuspid S′). The left ventricular diastolic function will be evaluated by the relationship between the E wave of the mitral flow and the E wave of the mitral annulus on tissue Doppler (E/E′ ratio) and by the diastolic velocity of the lateral mitral annulus (lateral E′).

All participants will be submitted to the Mini Mental State Examination (MMSE), an instrument developed by Folstein, Folstein, McHugh (1975), consisting of two parts, one covering orientation, memory, and attention (whose maximum score is 21 points) and the other about specific skills, such as naming and understanding (whose maximum score is 9 points), totaling a score of 30 points; higher values indicate greater cognitive performance. The score can be influenced by the individual’s education, following the proposal by Brucki et al. [5]: 20 points for illiterates, 25 points for people with 1 to 4 years of schooling, 26.5 for 5 to 8 years, 28 for those aged 9 to 11 years, and 29 for those aged over 11 years. Those with lower scores in their education category will be excluded from the study.

### Primary outcome measurements

The primary outcome is functional performance.

#### Six-minute walk test (6MWT)

The 6MWT will be used to assess the functional capacity of the participants [7]. The 6MWT will be held in a flat corridor 30 m long. The subjects will be oriented and encouraged to walk as far as possible in a stretch marked with colored tapes on the ground, for a period of 6 min with standardized phrases. It will be emphasized that if there is a need to interrupt the route, you must do so. The test will be performed twice, with an interval of 30 min between them, according to the recommendations of the ATS (ATS Committee on Proficiency Standards for Clinical Pulmonary Function Laboratories, [1]). After that, the test with the best performance (distance covered in meters) will be selected for analysis. In each test, the subject will remain for 5 min at rest sitting, then the same time at rest in the orthostatic position, and in sequence, will start the exercise. Performance recording will be performed at the peak of the exercise, as well as the presence and duration of pauses during walking and desaturation >4%, if any. A recovery time of 6 min after the exercise will be considered. HR (Polar H10, Polar, Kemple, Finland), BP (BIC sphygmomanometer, Itupeva, São Paulo) by the auscultatory method (Littman Classic II stethoscope, USA), SpO_2_ by digital oximeter (Nonin, model 2500, Minneapolis, MN), and the modified scale of perceived exertion Borg CR-10 will be used and their values recorded in the moments of rest sitting pretest, exercise peak, and first, third, and sixth minutes of recovery.

### Secondary outcome measurements

Secondary outcomes are cardiopulmonary exercise capacity (CPX), inflammatory cytokines, and quality of life.

#### Cardiopulmonary exercise test

Physical capacity will be assessed by CPX on a CG-04 ergometric bicycle (Inbramed, Porto Alegre, Brazil) with data directed to the breath-to-breath ergospirometer (PNOE, ENDO Medical, CA, USA) with a ramp protocol of 5–10 watts per minute. The volunteer must use a mask and mouthpiece system in which there is a bidirectional digital volume transducer, that is very light and precise, and capable of measuring lung volumes. The subjects will be familiarized with the exercise bike and with the Borg CR10 scale. Volunteers will be encouraged to perform the test until exhaustion. The test will be carried out together with a cardiologist medical professional, so that it is possible to detect any cardiac arrhythmias as well as assistance in the event of any intercurrences and a trained physiotherapist. The protocol will consist of the following phases: (I) rest period of 5 min sitting on the bicycle, (II) warm-up with free load for 3 min, (III) incremental phase in the ramp protocol with a cadence of 60 rotations per minute, (IV) 1-min active recovery with free load, and (V) passive recovery of 5 min standing on the bicycle. A 12-lead electrocardiogram (ECG) will be continuously monitored during the test, and blood pressure will also be recorded before the test, every 3 min during exercise and in the recovery period. The Borg CR-10 dyspnea scale will also be used to assess muscle fatigue in the lower limbs and shortness of breath during the test. The test will be interrupted if the patient presents physical exhaustion and/or presents one or more of the following criteria: (I) maximum/leg fatigue or dyspnea; (II) angina or electrocardiographic evidence of ischemia or malignant arrhythmia (ventricular tachyarrhythmia, bigeminy, and [atrioventricular and branch] blocks); (III) pressure changes with an increase greater than 220 mmHg in systole and above 100 mmHg in diastole; (IV) SpO_2_ drops to values ≤ 88%; and (V) patient request interruption of the test. Subjects will be encouraged to reach the maximum capacity limited by symptoms, characterized by a *V̇*CO_2_/*V̇*O_2_ ratio (RER) ≥ 1.1. The metabolic variables will be measured by the PNOE ergospirometer (ENDO Medical, CA, USA), and the gas analysis for the primary outcome will measure the absolute peak *V̇*O_2_ (mL/min), the corrected peak *V̇*O_2_ (mL/kg/min) and production of carbon dioxide (*V̇*CO_2_, mL/min). The variables slope of minute ventilation by CO_2_ exhalation (*V̇*E/*V̇*CO_2_ slope) and O_2_ consumption efficiency (OUES) will be evaluated during the cardiopulmonary test and before and after aerobic training. For variable calculations, the *V̇*E/*V̇*CO_2_ slope will be obtained by linear regression analysis of the relationship between *V̇*E and *V̇*CO_2_. Additionally, the relationship between *V̇*O_2_ and *V̇*E, expressed by the efficiency of oxygen consumption (OUES), will be determined by the logarithmic curve using the following equation: *V̇*O_2_ =a*log *V̇*E + b, where the constant “a” represents the rate of increase of V̇O2 in response to *V̇*E increment (Myers et al., [22]).

#### Inflammatory profile analysis

The inflammatory profile evaluation, including IL-1β, IL-6, IL-12p70, and TNF, which have a pro-inflammatory profile, and IL-10, which has an anti-inflammatory profile, was quantified using cytometric bead arrays (BD™ Human Inflammatory Cytokine kit (BD Biosciences, San Diego, CA, USA), according to the manufacturer’s instructions. Samples were acquired in a FACSCanto II (BD Biosciences, San Jose, CA, USA), and FCAP-Array software v3 (BD Biosciences, San Jose, CA, USA) was used for data analysis. Data were reported in picograms per milliliter (pg/mL) concentrations, according to the standard curves provided in the kits.

The pre- and post-assessment will be performed at the same hour, in a room with a controlled temperature (18 to 22 °C).

#### Quality of Life Questionnaire

The Minnesota Living with Heart Failure Questionnaire (MLHFQ) is an important tool to assess the limitations that are often associated with how much heart failure prevents patients from living as they would like. It is presented as the most-used instrument internationally and has great reliability, it was developed specifically for heart failure and consists of 21 questions [9]. The response scale for each question ranges from 0 (no) to 5 (too much), where 0 represents no limitations and 5 represents maximum limitations. These questions involve a physical dimension (from 1 to 7, 12, and 13) that is highly interrelated with dyspnea and fatigue, an emotional dimension (from 17 to 21) and a general dimension, related to financial considerations, medication side effects, and lifestyle (numbered 8, 9, 10, 11, 14, 15 and 16) which, added to the previous dimensions, form the total score. The last month should be considered to answer the questions.

### Intervention

Clinical assessments will be performed at baseline (T0) and at the end of 4 weeks (T1), consisting of a symptom-limited cardiopulmonary test, 6MWT, quality of life assessment, and laboratory tests (Table 1).

**Table 1:**
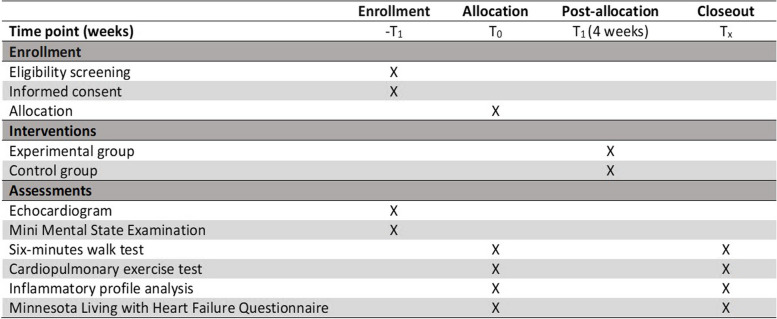
Schedule of enrollment, interventions, and assessments

*−T*_*1*_, baseline assessment before randomization; *T*_*0*_, allocation; *T*_*1*_, period of interventions; *T*_*x*_, analysis of interventions

The experimental group will be submitted to anodal tDCS over the left temporal region corresponding to the T3 area according to the International 10/20 Electroencephalogram system, and the cathode will be positioned over the right supraorbital area to modulate the insular cortex during continuous aerobic exercise with a constant current intensity of 2 mA for 20 min in each session. The control group will undergo sham tDCS during continuous aerobic exercise, with electrodes positioned in identical regions to the experimental group. To ensure blinding, an envelope will be made available with a numerical sequence to be used in the device for applying tDCS, whose study mode allows active stimulation during the 20 min of intervention (experimental group) or active stimulation only for a few seconds and cessation of stimulation (control group). Thus, participants, evaluators, and applicators of the research protocols will be blinded. Although no current is applied, sham stimulation mimics sensations from active tDCS, corroborating the blinding of the study. They all will perform three times/week, for a total of 12 sessions in 4 weeks.

Each aerobic exercise session will consist of three phases: warm-up, continuous aerobic exercise, and cool-down. The warm-up will be performed on an ergometric bicycle for 5 min with free load. Continuous aerobic exercise will be performed on an ergometric bicycle, as follows: during the first week, exercises will be performed for 15 min, workload will be selected by Borg 3–4 and/or an anaerobic threshold; for the next 3 weeks, the workload will be selected by Borg 3–4 and/or 60–70% peak HR in cardiopulmonary exercise test (HR will be used for training safety), for 25–30 minutes, maintaining rotation of 60 rpm every week. Cooling down will also be performed on the ergometric bicycle for 10 min with a free load (Table 2).

**Table 2:** Frequency, intensity, time, and type (FITT) of aerobic training

Phase	Frequency	Intensity	Time	Type
Warm-up	3 days/week	Free load	5 min	Ergometer bicycle
		Week 1: Borg 3–4 and/or anaerobic threshold	15 min	Ergometer bicycle
Continuous aerobic exercise	3 days/week			
		Weeks 2–4: Borg 3–4 and/or 60–70% peak HR in CPX	25–30 min	Ergometer bicycle
Cool-down	3 days/week	Free load	10 min	Ergometer bicycle

*HR* Heart rate, *CPX* Cardiopulmonary exercise test

In all sessions, the following will be monitored: heart rate by heart rate monitor (Polar H10, Polar, Kempele, Finland), partial oxygen saturation (SpO_2_) by pulse oximeter, and perceived exertion, measured by the Borg scale CR-10 every minute, which will be a criterion for increasing the load in training, as well as for interruption.

There are no restrictions regarding concurrent care during the trial. Furthermore, it is emphasized that patients will receive concurrent clinical treatment alongside physical training, and any changes in dosage or medication alteration will be recorded.

### Criteria for discontinuation

The criteria for discontinuation are worsening of symptoms, clinical instability, and emergence of serious infectious or viral diseases that are difficult to control.

### Sample size

For the sample size estimation, the following main outcome was *the* distance covered in the 6MWT. As there are no studies that have previously evaluated these outcomes, we used Du et al. [7] as the parameter of the minimal clinically important difference (MCID) value of 6 WMT in HF patients. The increase of 30 m was described as the mean value among studies. To calculate the effect size in the present study, we accepted 30 m of physical capacity improvement as the MCID to the control group (sham-tDCS during aerobic exercise) and, empirically, we chose 45 m of physical capacity improvement as the MCID to the experimental group (anodal tDCS during aerobic exercise). The effect size calculation was 0.41 Cohen’s *F* value. Therefore, we used the following parameters as sample size calculations for the ANOVA two-way test between factors: effect size of 0.41 by Cohen’s *F*, *α* = 5%, *β* = 80%, 2 groups and 2 measurements, and correction between measurements of 0.5. A total of 38 volunteers were reached,however, with the possibility of sample loss of 20%, a sample of 44 participants was estimated, 22 for the experimental group and 22 for the control group. For the calculation, the statistical program G*Power 3.1 (University of Dusseldorf, Dusseldorf, Germany) was used.

### Recruitment

We will use the expanded form of recruitment, by free demand, through dissemination in the media (ads on social networks, radio, and television), from which we will be able to have user representatives from different health units. We will visit medical units that are references to treatment for heart failure in the city of Manaus, Amazonas, and will deliver flyers with information about the project, and telephone numbers to contact the researchers. Once the eligibility criteria are met, they will be able to participate in the study.

### Strategies for trial retention

At the beginning of the training, all participants will be instructed on the importance of exercises for their health as a medical education strategy, as well as to encourage adherence to the program. The day before each session, every participant will receive a reminder message via cell phone confirming the date and time. Absences can be circumvented by rearranging the schedules within the same week.

### Randomization

The randomization list will be generated electronically by group statisticians, with a random number of blocks consisting of 4 to 6 participants per block. The randomization list will be generated in the statistical software R version 3.6.3 (Project R for statistical computing), using the blockrand package. The randomization process will be carried out by an external researcher not involved in the study, who will generate the randomization list on the computer. The same investigator will be responsible for maintaining and protecting allocation confidentiality and, in addition, will keep a copy of the list in a place inaccessible to participants and research staff. Participants will be allocated to intervention groups through codes generated by the software. To avoid possible errors and misguidance, random numbers will be documented with “I” for intervention and “C” for control. This process will be recorded to ensure that the envelopes are sealed with the respective names of the participants. All envelopes will be coated with aluminum foil to prevent possible reads through transparency. The allocation sequence will be blinded to researchers, investigators, and study participants. The allocation of participants to the intervention groups will only be revealed after the individual signs the informed consent, thus ensuring that the status of the group is not known during the study. In case of risk to the patient, the masking will be undone.

### Statistical analysis

Exploratory analyses will be carried out allowing a survey of the frequency distribution and calculation of means and standard deviations. The 95% confidence intervals will be considered, and effect sizes will be calculated for primary and secondary outcomes. Statistical significance will be stipulated with *p*<0.05. The Shapiro-Wilk test will be applied to analyze the normality of the sample distribution; unpaired *t*-test will be used for numeric variables, comparing means between two groups; and Fisher’s exact test will be used for categorical variables. Two-way ANOVA for between factors accompanied by post hoc Bonferroni analysis will be used to measure and predict the degree of relationship between inter- and intragroup variables. Pearson correlation will be performed to test the association between two continuous variables, and multiple linear regression will be used to quantify the relationship between predictor variables and the outcome variable. For data analysis, the statistical programs Jamovi (Jamovi Project, Sydney, Australia) and GraphPad Prism 7.0 (GraphPad, CA, USA) will be used. We will not perform sub-analysis, mainly because the primary stage is well defined in relation to the central question. Furthermore, it may be necessary to perform adjusted calculations for some variables, such as age, weight, BMI, EF%, and β-blocker drug dose. For this purpose, the utilization of these variables as covariates will be used in the ANOVA two-way analysis described above.

Participants who discontinue or deviate from intervention protocols will have their data on 6MWT and inflammatory cytokines collected.

### Data monitoring

Details of clinical site monitoring are documented in a Research Ethics Committee-approved clinical monitoring plan, which describes in detail who will perform monitoring, how often, and the distribution of monitoring reports.

An interim analysis and stopping committee were established at the request of the research ethics committee. This committee will meet regularly (every 3 months) to review the data and assess the study’s continuation. Nonetheless, any unforeseen adverse events that transpire during the intervention period will be promptly reported to a highly skilled physiotherapist who is not directly engaged in the trial’s execution. This physiotherapist will be accessible to make the ultimate decision to halt the trials in the event of unexpected harm.

All events must be managed and reported in compliance with all applicable regulations and included in the final clinical trial report. All physical/clinical symptoms described at each visit will be recorded as an adverse event if they occur or worsen during the study and are related to the study protocol.

### Access to data

If there is a need to release raw data due to any funding agency, scientific journal, or the ethics committee, upon request, the conclusive trial data for this protocol can be provided.

### Study organization and funding

This trial will be conducted according to relevant ethical frameworks and has received approval from the institutional ethical review board. No funding was received. The results will be submitted for publication in journals related to the area of cardiopulmonary rehabilitation, and access to the final trial dataset may be obtained from the authors upon reasonable request.

### The quality of the clinical trial team and the ongoing study protocol

The study will have a hierarchical relationship between the main study organization committee and the group responsible for analysis, assessments, recruitment, and training. In total, we will have an average of 14 researchers involved in the study, combining all parties. On Fridays, we will hold meetings to align potential adjustments to improve the quality of the protocol’s progress. Patient-related issues regarding the research development will also be addressed. We will also have periodic meetings of the evaluation and training groups to consider improvements in the volunteer-evaluator and volunteer-physiotherapist interaction to ensure the highest quality training and respect for study subjects. This will help achieve the best possible performance for an ethical, high-quality, replicable study with internal validation.

## Discussion

HF is a major health problem linked to heavy drug consumption resources and medical costs. Although the incidence has remained stable and even declined slightly over time, the prevalence is increasing improving HF treatment and increasing the life expectancy of the population. Despite HF treatment, morbidity and the death rate remain high [23, 29]. Exercise intolerance is a characteristic symptom of HF and is associated with increased disability and mortality. Sedentary lifestyle leads to decreased peak *V̇*O_2_ and poor quality of life in patients with heart failure [6, 26].

Although previous studies have investigated the combined effect of tDCS on T3 area and physical performance and have suggested that tDCS could have reduced ratings of perceived exertion by affecting the activity of the IC, and therefore increase exercise tolerance [20], Okano et al, [24]; [12], different methodologies were applied, especially with regard to characteristics of the participants and type of exercises. The present study is the first to evaluate the effects of the addition of anodal tDCS to aerobic exercise training for improving physical and functional performance, decreasing perceived exertion, altering the quantification of inflammatory cytokines, and improving the subclinical values of the cardiopulmonary test in patients with HFrEF, which could help clinicians in their decision-making process about novel portable devices currently available, such as noninvasive brain stimulation.

This double-blind randomized controlled trial has some limitations. The experimental and control interventions consist of aerobic exercises performed three times per week over 4 weeks and, therefore, depend on participants’ motivation, adherence, and commitment. Strategies to encourage participants to comply with the protocol are planned as described previously.

In conclusion, the results of this trial may result in an important advance in cardiac rehabilitation for patients with chronic HF. First, an adjunct intervention may help improve exercise capacity in patients with HFrEF. Second, if exercise tolerance is enhanced, the benefits may be accompanied by a better quality of life. Individuals may experience greater performance in activities of daily living, increased social interactions, and increased ability to engage in work and leisure activities, which is a desirable goal for both patients and rehabilitation professionals.

## Trial status

The present trial protocol corresponds to version 3.0, April 2023, and it is in the recruiting phase. Recruitment will be started in September 2023. It is expected to be complete in 36 months, by September 2025. Trial registration: ReBEC - RBR-10w787j6. Registered on 25 April 2023

## Ethics and dissemination

The study protocol was approved by the Ethics Committee of the Federal University of Amazonas (Universidade Federal do Amazonas), Brazil, and followed the norms of the World’s Association Declaration of Helsinki. All patients with HF recruited in the ELETRIC study gave their informed consent at the time of enrollment. All source records, including electronic data, will be stored on secure systems in accordance with institutional policies and federal regulations. Names or identifications will not be released unless the safety oversight committee approves and aligns with consent or in accordance with laws for required reporting. The results of this study will be disseminated in peer-reviewed journals and academic conferences, including the full protocol that was submitted for publication.
